# Case Report: A case of neuropsychiatric lupus with primary central nervous system diffuse large B-cell lymphoma

**DOI:** 10.3389/fimmu.2025.1636597

**Published:** 2025-08-21

**Authors:** Yuexi Zhang, Shaoling Zheng, Yiming Li, Shuyang Chen, Xin Guo, Zhixiang Huang, Weiming Deng, Yuheng Xing, Zhengping Huang, Tianwang Li

**Affiliations:** ^1^ Department of Rheumatology and Immunology, The Affiliated Guangdong Second Provincial General Hospital of Jinan University, Guangzhou, China; ^2^ Department of Pathology, The Affiliated Guangdong Second Provincial General Hospital of Jinan University, Guangzhou, China; ^3^ Department of Rheumatology and Immunology, Zhaoqing Central People’s Hospital, Zhaoqing, China

**Keywords:** neuropsychiatric systemic lupus erythematosus (NPSLE), primary diffuse large B-cell lymphoma of the central nervous system lymphoma (PCNSL), brain abscess, misdiagnosis, case report

## Abstract

**Background:**

The coexistence of neuropsychiatric systemic lupus erythematosus (NPSLE) and primary diffuse large B-cell lymphoma (DLBCL) of the central nervous system (CNS) (PCNS DLBCL) is extremely rare in clinical practice. This article retrospectively analyzes the clinical manifestations, imaging examinations, pathological diagnosis, and treatment process of a patient with NPSLE, from the appearance of intracranial abnormal signal shadows to the final diagnosis of PCNS DLBCL.

**Case summary:**

A 32-year-old Chinese female patient had previously visited our hospital due to vomiting and delirium and was diagnosed with NPSLE. In February 2021, she returned to our hospital with vomiting again. Laboratory tests revealed elevated infection markers and Epstein-Barr virus infection. Brain CT and MRI showed an abnormal intracranial lesion on the left side, which was initially considered to be a brain abscess. After one week of ineffective anti-infection treatment, the patient underwent surgery, during which the lesion was identified as a brain tumor and successfully resected. The final diagnosis was PCNS DLBCL. The patient improved after treatment and was discharged from the hospital. There has been no recurrence of NPSLE or lymphoma within three years.

**Conclusion:**

When patients with NPSLE develop new intracranial lesions, misdiagnosis is likely to occur. Imaging and pathology are crucial, and clarifying the nature of the lesion is conducive to a good long-term prognosis.

## Introduction

NPSLE is a severe complication of systemic lupus erythematosus (SLE) involving the CNS, with a high mortality rate. PCNS DLBCL is a type of non-Hodgkin lymphoma confined to the CNS, and its clinical symptoms are atypical (1), including headache, nausea, vomiting, limb weakness, etc., which makes it prone to misdiagnosis in the early stages. As reported, patients with neuropsychiatric lupus are susceptible to developing central nervous system lymphoma, which may be attributed to immune dysregulation or long-term immunosuppressive therapy. However, in most cases, the lymphoma is secondary and originates from metastasis from other sites, including the eye (especially primary vitreoretinal lymphoma), systemic lymphoma (such as involvement of lymph nodes, spleen, bone marrow, etc.), and the testis (2). The occurrence of PCNS DLBCL is extremely rare, which results in difficulties in diagnosis and a poor prognosis. Here, we report in detail the diagnosis and treatment process of a case of NPSLE complicated with PCNS DLBCL.

## Case report

In 2006, a 19-year-old female patient developed recurrent oral ulcers, malar rash, and arthralgia. Laboratory tests revealed ANA 1:100 with a speckled pattern, positive ds-DNA antibodies, low complement C3 and C4, and hemolytic anemia. Systemic lupus erythematosus (SLE) was diagnosed at an outside hospital. Treatment with oral glucocorticoids (initially 40 mg daily, then tapered) and hydroxychloroquine was initiated, leading to improvement of symptoms. The patient has since adhered to regular follow-up and management. In June 2020, she began experiencing symptoms of vomiting and delirium and was admitted to our hospital for treatment. Her cerebrospinal fluid (CSF) examination showed a white blood cell count of 53×10^6^/L, with a percentage of mononuclear cells of 94.3%. CSF pathogen tests were negative. Cranial MRI revealed multiple ischemic foci in the left centrum semiovale and the right corona radiata. The primary diagnoses were neuropsychiatric lupus. After treatment with intravenous methylprednisolone 40 mg for 5 days and human immunoglobulin 20 mg for 5 days, the patient’s condition stabilized. She was discharged and continued to take oral glucocorticoids, mycophenolate mofetil, and hydroxychloroquine sulfate regularly to control her condition.

**Figure 1:**
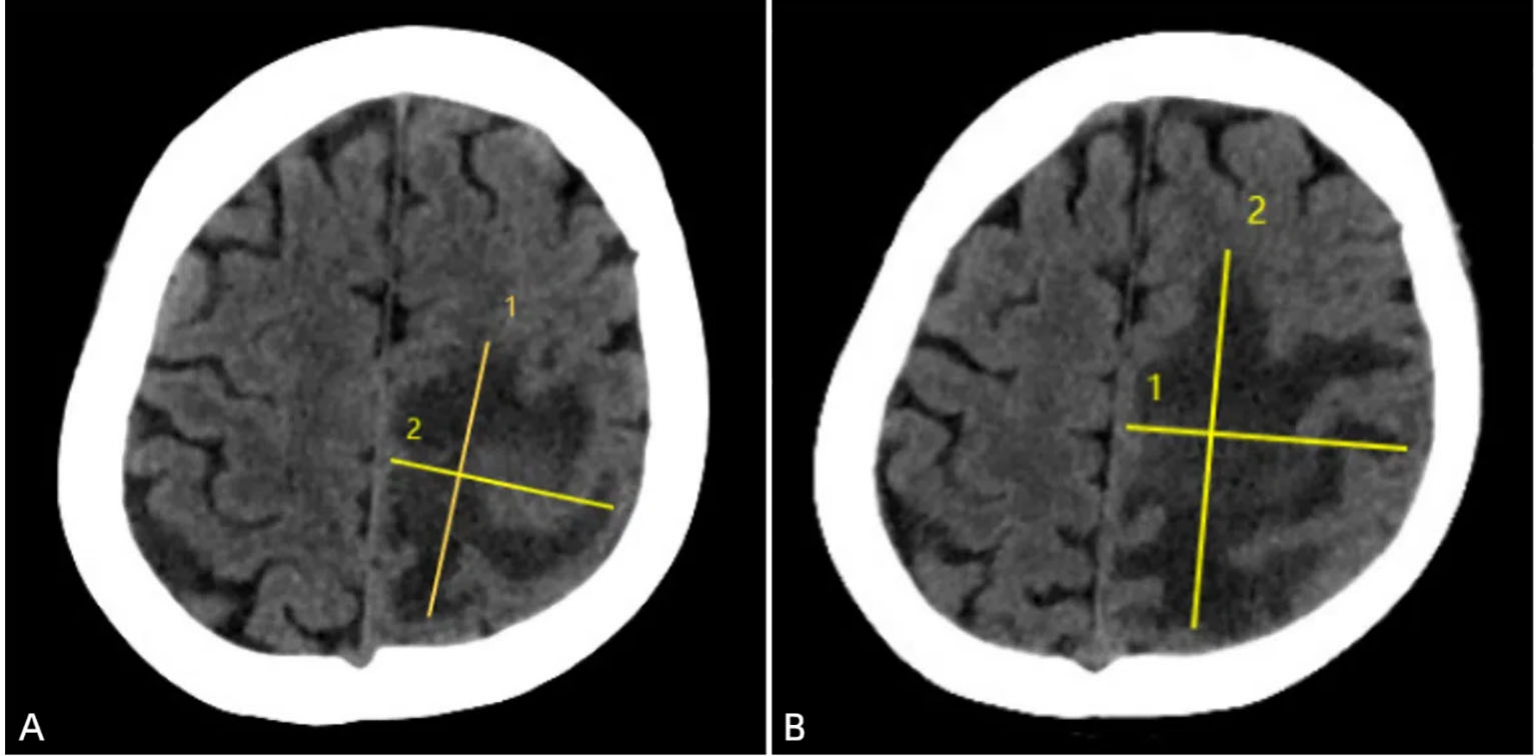
Brain CT upon admission (February 27, 2021) demonstrated a large hypodense area in the left parietal lobe, with clear boundaries, uneven density, and a maximum lesion size of approximately 55mm × 45mm. The left lateral ventricle was enlarged **(A)**. One week after anti-infective treatment (March 11, 2021), a follow-up CT scan revealed that the large hypodense area in the left frontal and parietal lobes had increased in size, with clear boundaries, uneven density, and a maximum lesion size of approximately 56mm × 76mm. A slightly higher density mass was seen within the lesion, and the left lateral ventricle was enlarged **(B)**.

**Figure 2:**
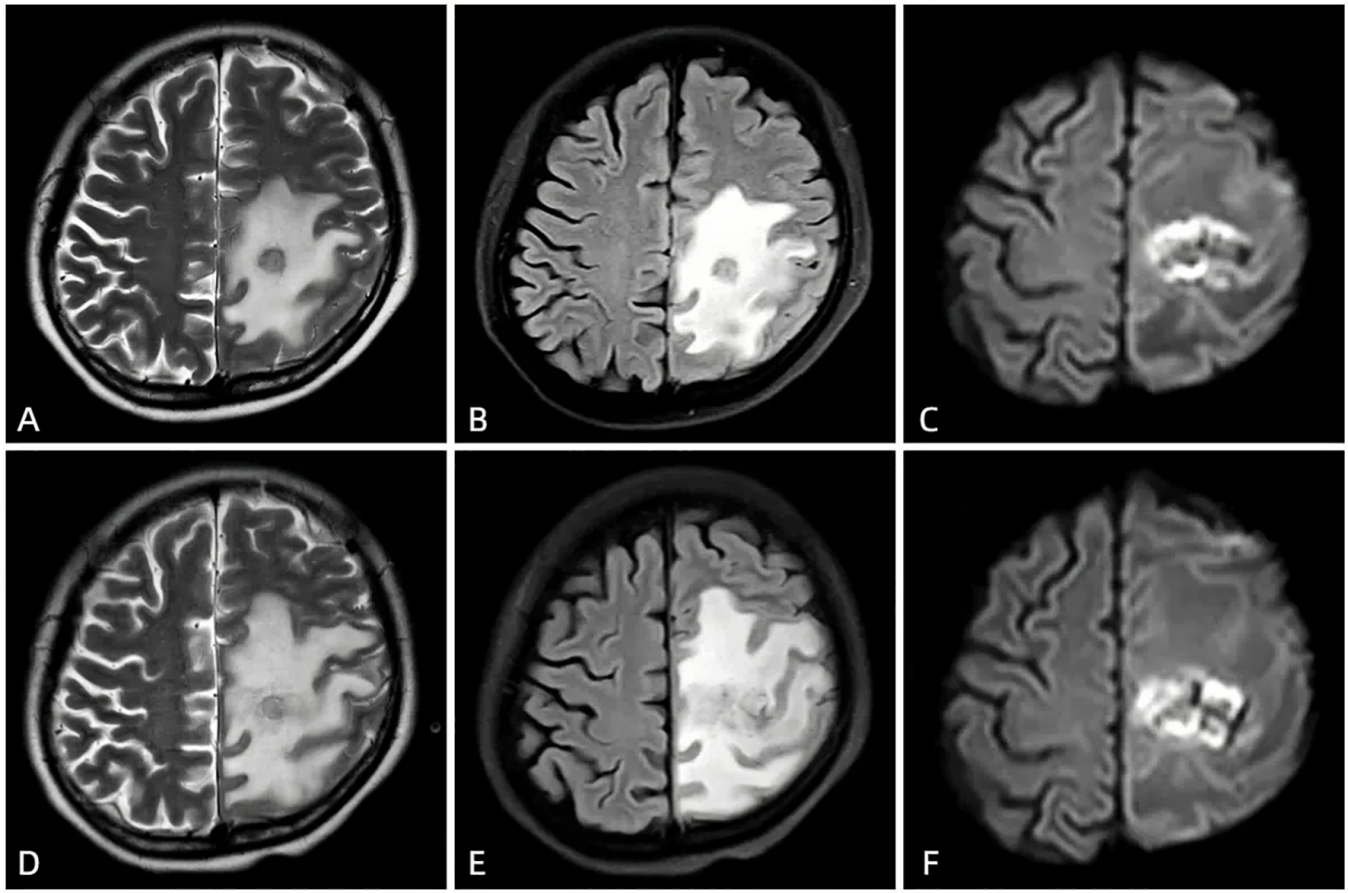
The first MRI after admission (March 4, 2021) showed a new round-like long T1 and slightly long T2 signal in the left frontal and parietal lobes **(A)**, slightly high signal on FLAIR **(B)**, ring-shaped high signal and central low signal on DWI **(C)**, and several low signals on ADC, involving the left lateral ventricle. The enhanced scan showed ring enhancement, with no obvious enhancement in the cystic cavity. After anti-infective treatment (March 14, 2021), the follow-up MRI showed symmetrical bilateral cerebral hemispheres, a round-like long T1 and slightly long T2 signal in the left frontal and parietal lobes **(D)**, slightly high signal on FLAIR **(E)**, ring-shaped high signal and central slightly low signal on DWI **(F)**. The lesion had slightly increased in size and involved the left lateral ventricle; large areas of long T1 and long T2 signal edema were seen around the lesion, with a slightly expanded range compared to before.

However, in February 2021, the patient experienced recurrent vomiting. Two weeks later, she suddenly developed weakness in her right limbs and was unable to walk. She was admitted to our hospital. Physical examination revealed decreased muscle strength in the right limbs (grade 2 muscle strength in the right upper limb and grade 3 muscle strength in the right lower limb), with positive findings on the right-sided neurological examination (Babinski sign and Chaddock sign). No other abnormalities were noted. Laboratory tests were detailed in **Table 1**. Neuroimaging (CT/MRI) revealed a hypodense lesion in the left parietal lobe (**Figures 1A**, **2A–C**). Echocardiography and abdominal ultrasound showed no significant abnormalities. Based on a comprehensive analysis of the patient’s symptoms, signs, and laboratory test results since the onset of this episode, we considered that the abnormal signals in the patient’s brain might be due to a brain abscess.

**Table 1:** Laboratory profile at admission demonstrating hematologic, inflammatory, and renal abnormalities in a patient with SLE and subsequent PCNS-DLBCL diagnosis.

Parameter	Value	Parameter	Value
Hemoglobin	71 g/L	Blood urea nitrogen	9.41 mmol/L
White blood cells	14.42×10^9^/L	Creatinine	328 μmol/L
Neutrophils	12.74×10^9^/L	24-hour urine protein	3.626 g/24h
Lymphocytes	0.69×10^9^/L	Direct antiglobulin test	Weakly positive
Platelets	331×10^9^/L	HIV/HBV/HCV/TB	Negative
Erythrocyte sedimentation rate	48 mm/h	ANA, anti-Sm, anti-dsDNA, ribosomal P	Normal
C-reactive protein	43.3 mg/L	CSF Protein	0.55 g/L
Procalcitonin	3.96 ng/mL	CSF Glucose	2.05 mmol/L
D-dimer	1.97 mg/L	CSF Cell count/chloride	Normal
Activated partial thromboplastin time	47.6 s	CSF High-throughput testing	Epstein-Barr virus
C3	0.693 g/L	CSF Cytopathology	No malignant cells
C4	0.248 g/L	CA125	72.82 U/mL

Therefore, we initiated empirical antibiotic therapy (including acyclovir and piperacillin-tazobactam). After one week of anti-infective treatment, the patient’s consciousness slightly improved, but there was no significant change in muscle strength, and white blood cells and neutrophils did not decrease significantly. A follow-up brain CT scan (performed on March 12, 2021) showed an increase in the size of the lesion (**Figure 1B**), and MRI also indicated an expansion of the edema surrounding the lesion in the left frontal and parietal lobes (**Figures 2D, F**). These findings suggested that the current treatment did not effectively control the progression of the disease. Notably, the abnormal brain signal on MRI showed a ring-enhancing pattern with a central low signal. In addition to a brain abscess, the possibility of a brain tumor could not be ruled out. For example, intracranial metastases and gliomas could form central necrosis and liquefaction, presenting as ring enhancement on CT and MRI. Therefore, to prevent further deterioration of the patient’s condition, a multidisciplinary consultation was convened, and we planned to perform a craniotomy to explore the exact nature of the lesion.

**Figure 3:**
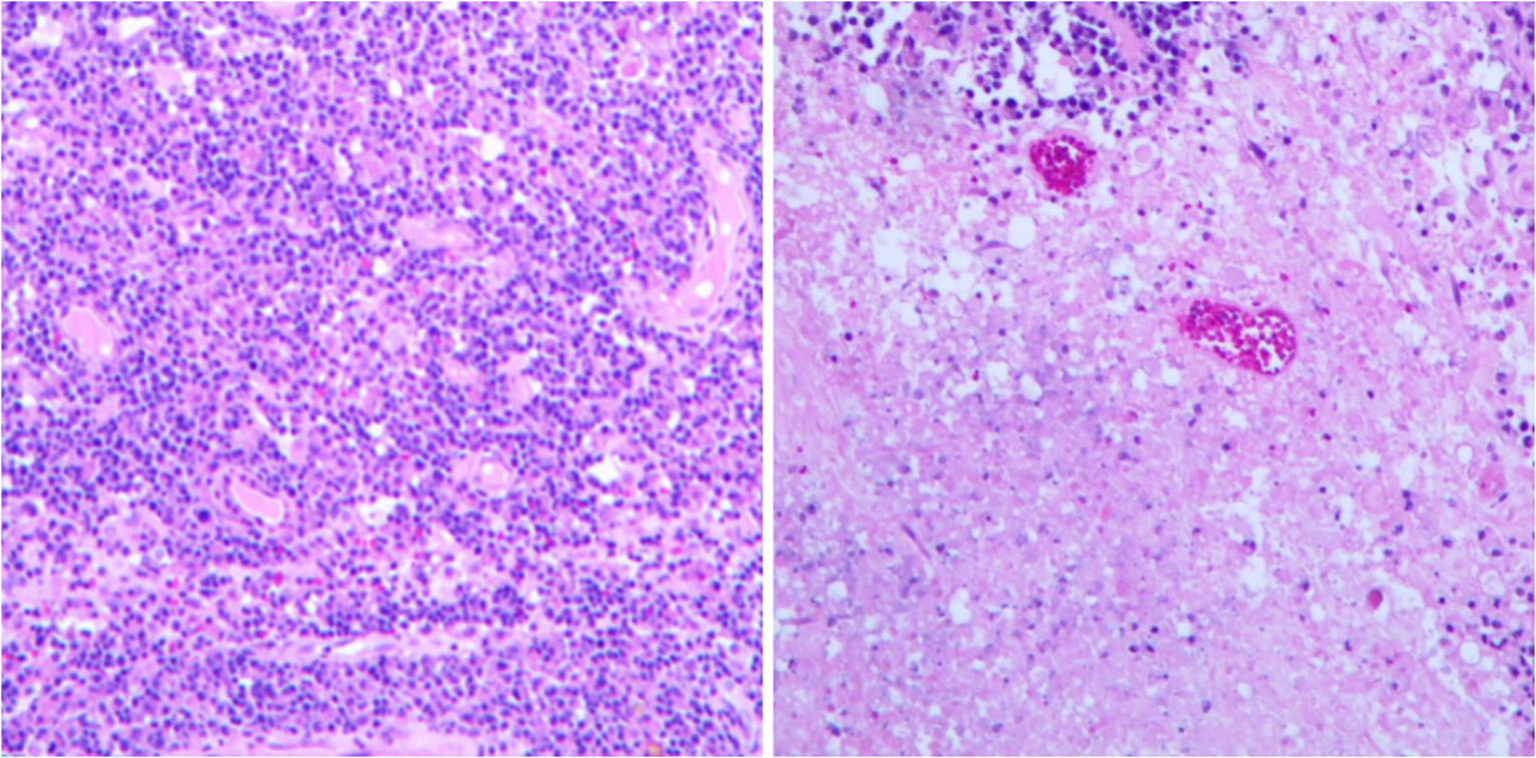
Histopathological examination showed extensive necrosis of the submitted brain tissue, with focal atypical lymphocytes proliferating around blood vessels. Hematoxylin and eosin staining; 10× magnification. (Immunohistochemistry results: Atypical lymphocytes were CK/pan (−), CD19 (+), CD20 (+), CD1a (−), S-100 (−), Ki-67 (approximately 70%+), GFAP (−), CD79a (+), MUM1 (mostly +), CD10 (−), BCL-2 (approximately 80%+), BCL-6 (a few +), c-Myc (approximately 20%+), P53 (partial, moderate-weak +); CD3 and CD5 (showing a few T lymphocytes +). Special staining results: Acid-fast (−), Gomori methenamine silver (−), fungal fluorescence staining (−), acid-fast bacillus fluorescence staining (−).

On March 2021, the patient underwent left parietal craniotomy for suspected brain abscess/tumor exploration. Intraoperative needle aspiration revealed no pus, and imaging confirmed the lesion as a brain tumor. Consequently, the procedure was changed to a Left Frontal and Parietal Brain Tumor Resection. The postoperative pathology revealed DLBCL (**Figure 3**). Subsequently, the patient underwent a whole-body PET-CT examination, which did not detect any lymphoma lesions in other parts of the body. The bone marrow biopsy also did not reveal any lymphoma. The patient was ultimately diagnosed with PCNS DLBCL. After the surgical removal of the lymphoma from the brain, the patient’s general condition was poor, and she was transferred to the ICU for close monitoring. When the condition stabilized, the patient continued to regularly use immunomodulatory drugs to manage her SLE.

**Figure 4:**
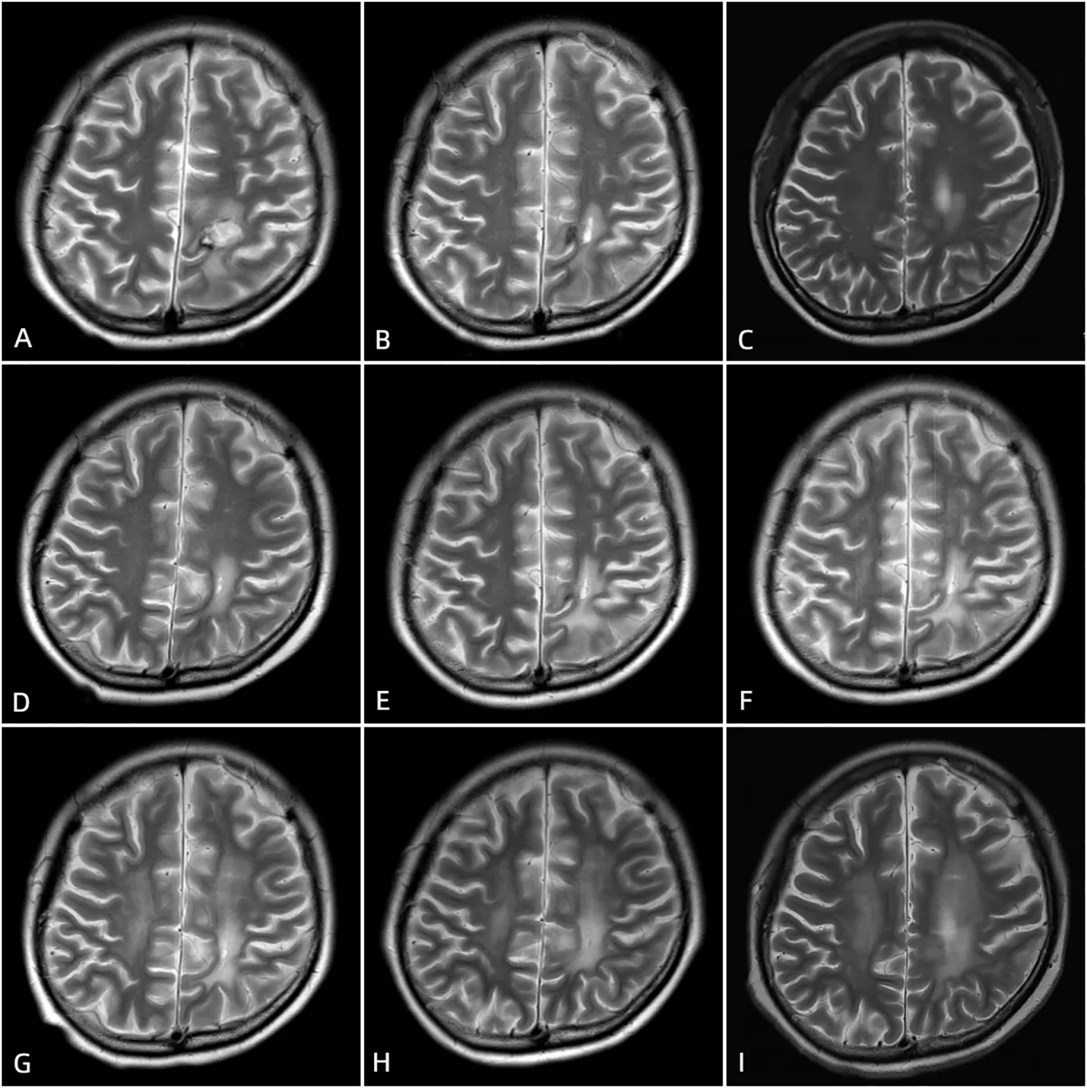
Postoperative cranial MRI scans after surgery, taken at 1 month **(A)**, 3 months **(B)**, 6 months **(C)**, 10 months **(D)**, 13 months **(E)**, 19 months **(F)**, 24 months **(G)**, 30 months **(H)**, and 40 months **(I)** postoperatively, show postoperative changes in the left frontal and parietal lobes lymphoma. The hemorrhagic foci in the surgical area gradually absorbed. On T1-weighted imaging (T1WI), the signal is predominant, while on T2-weighted imaging (T2WI), high signal intensity is observed. A ring-shaped long T1 and short T2 signal is visible at the edge of the surgical area.

Postoperatively, her condition improved with infection prophylaxis and immunomodulation, her condition gradually improved. Her consciousness became clearer, and she was able to stand independently and walk with assistance. Laboratory tests and physical examinations showed improvements (white blood cells and neutrophils returned to normal, C-reactive protein was slightly elevated, muscle strength in the right upper and lower limbs was grade 4, and all neurological pathological signs were negative). She was eventually discharged on April 1, 2021, and in the same month, she was admitted to the oncology department of our hospital to complete postoperative whole-brain radiotherapy (WBRT, with a total dose of 36 Gy). During the 3-year follow-up after discharge, she regularly took Hydroxychloroquine 0.2g twice daily and Prednisone Acetate tablet 5mg once daily to maintain her condition. To date, her lymphoma has not recurred, and her NPSLE remains stable (**Figure 4**).

## Discussion

This case report describes the diagnostic and therapeutic process of a patient with NPSLE who was initially misdiagnosed with a brain abscess due to a new intracranial lesion, and was ultimately diagnosed with PCNS DLBCL. The coexistence of NPSLE and PCNS DLBCL is extremely rare, with only one case previously reported in Japan (3).

The patient’s initial presentation with vomiting and right limb weakness, alongside ring-enhancing MRI lesions, was highly suggestive of a brain abscess given her immunosuppressed state. However, the lack of response to antimicrobials and the lesion’s progression despite immunosuppression reduction raised suspicion for malignancy. This mimics NPSLE flares or infections but diverged critically in its radiological evolution: PCNS DLBCL often shows solitary, homogeneously enhancing lesions, but our case exhibited ring enhancement—a feature more typical of abscesses or metastatic tumors. The misdiagnosis emphasizes that atypical imaging patterns in NPSLE warrant early biopsy when empirical therapy fails.

In this case, the patient has a long history of SLE and was diagnosed with neuropsychiatric lupus and lupus nephritis at our hospital seven months ago (4, 5). After discharge, she regularly used corticosteroids and immunosuppressive agents to control her condition, and her disease remained stable for seven months. Previous literature has shown a certain correlation between the use of mycophenolate mofetil and primary central nervous system lymphoma (PCNSL) (3, 6, 7). Some previous studies have also indicated that other immunosuppressive agents, including azathioprine, methotrexate, and high accumulations of corticosteroids, can increase the risk of lymphoma (8–13). After only a seven-month stable period, her symptoms recurred and worsened, and cranial imaging revealed a new abnormal lesion. Therefore, the possibility of the long-term use of immunosuppressive agents predisposing to lymphoma could not be ruled out.

The patient’s journey from presenting with abnormal psychiatric symptoms and new intracranial lesions to being definitively diagnosed with PCNS DLBCL was a tortuous one. Although she had previously tested positive for antinuclear antibodies (ANA 1:100) on multiple occasions, during her treatment at our hospital, her ANA and other autoantibodies tested negative. It is relatively rare for patients with NPSLE to have negative ANA and other autoimmune antibodies. Possible reasons may include the use of immunosuppressive agents, improvement in the condition, and methodological differences in laboratory interpretation of low-titer antibodies. Studies also indicated that there are indeed a number of patients who do not exhibit positive antibodies during the course of lupus (14). Long-term follow-up is warranted to exclude false negativity.

This case illustrates that PCNS DLBCL should be considered in NPSLE patients with refractory neurological deficits and atypical imaging, even without systemic lymphoma. Early biopsy and multidisciplinary collaboration are critical. The paradoxical role of immunosuppression—both as a risk factor and a controllable variable—warrants further study in SLE-associated malignancies.

## Data Availability

The original contributions presented in the study are included in the article/supplementary material. Further inquiries can be directed to the corresponding authors.
